# Immunostimulatory Endogenous Nucleic Acids Perpetuate Interface Dermatitis—Translation of Pathogenic Fundamentals Into an *In Vitro* Model

**DOI:** 10.3389/fimmu.2020.622511

**Published:** 2021-01-11

**Authors:** Christine Braegelmann, Tanja Fetter, Dennis Niebel, Lara Dietz, Thomas Bieber, Joerg Wenzel

**Affiliations:** Department of Dermatology and Allergy, University Hospital Bonn, Bonn, Germany

**Keywords:** interface dermatitis/lichenoid tissue reaction, nucleic acid sensing, damage associated molecular patterns (DAMPs), type I immunity, lupus erythematosus, lichen planus, dermatomyositis, *in vitro* model

## Abstract

Interface dermatitis is a histopathological pattern mirroring a distinct cytotoxic immune response shared by a number of clinically diverse inflammatory skin diseases amongst which lichen planus and cutaneous lupus erythematosus are considered prototypic. Interface dermatitis is characterized by pronounced cytotoxic immune cell infiltration and necroptotic keratinocytes at the dermoepidermal junction. The initial inflammatory reaction is established by cytotoxic immune cells that express CXC chemokine receptor 3 and lesional keratinocytes that produce corresponding ligands, CXC motif ligands 9/10/11, recruiting the effector cells to the site of inflammation. During the resulting anti-epithelial attack, endogenous immune complexes and nucleic acids are released from perishing keratinocytes, which are then perceived by the innate immune system as danger signals. Keratinocytes express a distinct signature of pattern recognition receptors and binding of endogenous nucleic acid motifs to these receptors results in interferon-mediated immune responses and further enhancement of CXC chemokine receptor 3 ligand production. In this perspective article, we will discuss the role of innate nucleic acid sensing as a common mechanism in the perpetuation of clinically heterogeneous diseases featuring interface dermatitis based on own data and a review of the literature. Furthermore, we will introduce a keratinocyte-specific *in vitro* model of interface dermatitis as follows: Stimulation of human keratinocytes with endogenous nucleic acids alone and in combination with interferon gamma leads to pronounced production of distinct cytokines, which are essential in the pathogenesis of interface dermatitis. This experimental approach bears the capability to investigate potential therapeutics in this group of diseases with unmet medical need.

## Introduction

Interface dermatitis (ID), also referred to as lichenoid tissue reaction, describes a histopathological pattern defined by morphological anomalies of the epidermal basal cell layer characterized by perishing keratinocytes labeled vacuolar or hydropic colloid bodies. Anti-epithelial activity of autoreactive cytotoxic lymphocytes is causative (1). The distinct pattern is observed in clinically heterogeneous skin diseases including autoimmune skin disorders [lichen planus (LP), lichen sclerosus (LS), cutaneous lupus erythematosus (CLE), dermatomyositis (DM)] and immunologic reactions against viruses, drugs and specific tumors (“lichenoid” keratosis) (2, 3). In 1995, for the first time, Fäh et al. detected MxA expression not only in virally infected tissue but also in dermatoses featuring ID (4). These findings are explained by MxA expression being directly induced by type-I and type-III IFNs (5). Today, it is accepted that activation of the interferon system resulting in a cellular immune response is a pathogenic key feature of the histologic “look-alikes” sharing ID (1).

Amongst the autoimmune skin disorders featuring ID, LP, and CLE are considered prototypic (6): LP may affect the skin including its appendages and both oral and genital mucosa (7). Classical LP presents with violaceous papules generally accompanied by extensive pruritus. When affecting the nails, thinning, scarring, and even complete loss of the nail is possible. Lichen planopilaris affecting the hair follicles may cause scarring and permanent baldness. Affected mucosa usually presents as erosive (8). The clinical spectrum of lupus erythematosus is broad ranging from systemic manifestations [systemic lupus erythematosus (SLE)] to manifestations solely affecting the skin. Cutaneous lupus erythematosus (CLE) may present itself as one symptom of SLE or may occur as an isolated skin disease (9, 10). CLE manifestation can be further subdivided into four main subsets (acute, subacute, intermittent, or chronic). Acute CLE may present either with a localized facial indurated erythematous lesion (malar rash) or with a widespread erythematous maculopapular rash. Subacute CLE is characterized by either annular/polycyclic or by papulosquamous skin lesions. Intermittent CLE shows non-scaling and non-scarring skin lesions. The last subset is chronic CLE which may be further subdivided into chronic discoid lupus erythematosus, chilblain lupus and lupus erythematosus profundus. Chronic discoid lupus erythematosus constitutes the largest group and features scarring erythrosquamous plaques in a disc-like shape. Chilblain lupus is a rare acral variant of chronic CLE whereas lupus erythematosus profundus affects the subcutaneous fat (11).

Despite clinical heterogeneity and although the initial stimulus may differ between diseases featuring ID, final common path is a cytotoxic anti-epithelial directed attack by autoreactive T lymphocytes (12–14) that are recruited to the site of inflammation by keratinocytes producing large amounts of C-X-C Motif Chemokine Ligands 9/10/11 (CXCL9/10/11) (15).

We herein summarize etiopathological mechanisms involved in ID and particularly outline the role of innate nucleic acid sensing in keratinocytes as a hallmark of perpetuation of the proceeding “pro-inflammatory vicious circle”. We, furthermore, present a human *in vitro* model that functions as a tool to evaluate potential therapeutic interventions and thus facilitate prediction of therapeutic response to novel treatment strategies in diseases featuring ID.

## Interface Dermatitis—The Pathogenic Background

### Interferon Signaling and Cellular Response in Interface Dermatitis

A multitude of genes is differentially expressed similarly in both LP and CLE skin when compared to healthy skin. In particular, distinct associations have been described for genes concerning interferon signaling as well as associated downstream cascades (16–18). The type-I [IFNalpha(a)/beta(b)/kappa(k)] (1, 19–21) and type-III interferon system [IFNlambda(λ)] (22) do not only participate in antiviral immune defense, but also play an important pathophysiological role in ID. Particularly, they are expressed by respective lesional keratinocytes. Via autocrine loops, IFNs bind to their corresponding receptor on keratinocytes and unleash pro-inflammatory downstream cascades *via* activation of the JAK-STAT pathway (23–25). Finally, inflamed keratinocytes express CXCL9 (22), CXCL10 (26, 27) and CXCL11 (28). The corresponding CXCR3 receptors are expressed on activated pDCs (29), T cells [Th1-type CD4+ T cells (30) and effector CD8+ T cells (31–33)] and macrophages (34, 35). Hereby attracted pDCs contribute to the inflammation *via* further type-I interferon production (36, 37) and Th1 lymphocytes create a specific inflammatory milieu *via* secretion of distinct cytokines. T cells isolated from lesional skin of LP and CLE patients revealed high frequency of IFNy (IFN gamma) and TNFa, two key cytokines of Th1 lymphocytes (16). The type-II interferon, IFNy, sparks downstream cascades which partly overlap with those of type-I interferons (38): It induces CXCR3 ligands and the differentiation of naïve T cells into Th1 cells and it activates macrophages (39). In response to stimulation with IFNy and TLR (Toll Like Receptor) ligands macrophages undergo classical “M1” activation (40): This pro-inflammatory M1 phenotype is prevalently seen in rheumatic diseases (41) and has specifically been described in lichen planus (42) and lupus skin (43). Cytotoxic lymphocytes represent the last group of CXCR3 receptor carrying immune cells and execute their anti-epidermal attack *via* cytotoxic granules and the perforine/granzyme pathway (44). Apart from upregulation of genes mediating direct cytotoxicity, enhanced expression of markers of apoptosis (FASL) and necroptosis (RIP3) have been detected in ID (16).

### Nucleic Acid Sensing Induces Interferon Response and Mediates Inflammasome Activation as Well as Cell Death Cascades

Inflammatory cell death upon cytotoxic attack inevitably results in release of intracellular components, amongst them are endogenous nucleic acids (eNA). Nucleic acid sensing by the innate immune system functions *via* pattern recognition receptors (PRRs), that are activated by pathogen associated molecular patterns (PAMPs) or host molecules (damage associated molecular patterns, DAMPs) (45). Physiologically, PRRs enable sufficient immune response to either an invading pathogen or damage of host cells (46, 47). Sensing of self-RNA and self-DNA, however, also holds the potential to contribute to autoimmunity (45, 48, 49). In ID, the pro-inflammatory capacity of released nucleic acids is supposedly supported by the cathelicidin LL37 which has been shown to be overexpressed in CLE (50, 51), LP (52), and DM (53). Its complex formation with nucleic acids has been proven to enable transport of extracellular nucleic acid fragments into intracellular compartments (54). Specifically, our working group has previously shown that addition of LL37 enhances immunogenicity of nucleic acids in keratinocytes *in vitro* (50). Key features of downstream signaling of PRRs include induction of interferons (45, 46, 55) and inflammasome activation (56) as well as programmed cell death cascades (46).

The respective downstream mechanisms of important PRRs are as follows:

AIM2 (Absent In Melanoma 2) activates the inflammasome upon double stranded (ds) DNA sensing (57–59) which leads to Caspase 1 cleavage and finally maturation of the pro-inflammatory interleukins IL18 and IL1ß (60). Furthermore, activated Caspase 1 cleaves Gasdermin D which executes pyroptosis *via* pore formation in affected cell membranes (61). AIM2 is upregulated in skin samples of lichen planus patients (62). Inflammasome activity is enhanced in lupus erythematosus (63) and the inflammasome-activated cytokine IL18 is highly upregulated in the epidermis of CLE patients (64). The discovery of its dysregulation in autoimmunity suggests inhibition of inflammasome components as an interesting therapeutic approach, as postulated by Kahlenberg et al. (65).

Upon DNA binding to cGAS (Cyclic GMP-AMP synthase) an IFN response is unleashed (66): CGAS activates STING (Stimulator of IFN genes) (67, 68) which, in turn, interacts with TBK1 (TANK-binding kinase) resulting in phosphorylation of IRF3 (Interferon Regulatory Factor) and finally type-I interferon gene transcription (69). Furthermore, the cGAS-STING pathway has multiple functions in mediating cell death that are not fully elucidated, yet (46). Its activation by self-DNA is described as an important mechanism in autoimmunity which might constitute another promising target for therapeutic intervention (49).

IFI16 (Gamma-interferon-inducible protein 16) is a further key DNA sensor in human keratinocytes. It cooperates with cGAS in the activation of STING (70). Excessive IFI16-dependent production of IFN-I is considered an important mediator of autoimmune inflammation (71, 72) and has specifically been shown to contribute to SLE (73) and to cytokine induction in keratinocytes (74). Apart from STING-dependent type-I IFN production, IFI16 enables direct inflammasome activation (75, 76).

ZBP1 (Z-DNA Binding Protein) binds to ds nucleic acids when presenting in the unusual Z‐conformation (77). Activated ZBP1 recruits TBK1 and IRF3 (78) and triggers RIP3-dependent necroptosis (79) as well as NLRP3-dependent inflammasome activation (80). Specifically, aberrant sensing of endogenous nucleic acids by ZBP1 has been shown to induce inflammation in murine skin (81). Guo et al. have shown that ZBP1 activation also induces necroptosis in human cells (82) and sera from some SLE patients exhibit anti‐Z‐DNA autoantibodies (83). Thus, ZBP1 has been suggested as a potential therapeutic target that requires further research (84).

RIG-I-like receptors (RLRs) comprise three important sensors: RIG-I, MDA5 (Melanoma differentiation-associated protein), and LGP2 (Laboratory of Genetics and Physiology 2) (85) with the latter being considered a regulator of the others. RIG-I and MDA5 experience conformational changes upon cytosolic RNA sensing that result in exposure of their CARD domain and consecutive activation of IRF3 *via* phosphorylation of TBK1 and NFkB activation (85, 86). RLRs are also implicated in apoptosis and RIP3-mediated necroptosis (87, 88). Human keratinocytes constitutively express RIG-I and MDA5 (89). Challenge with IFNy or TNFa has induced RIG-I in a human keratinocyte cell line (90) and both RIG-I and MDA5 expression is increased in psoriatic skin (90). A specific single nucleotide polymorphism in the gene encoding MDA5 has been identified in autoimmune diseases, including SLE (91, 92).

Nucleic-acid-sensing TLRs are mainly expressed in the endosomes (93) and comprise TLR3 [recognizes dsRNA (94)], TLR7 and TLR8 [recognize ssRNA (95)] and TLR9 [recognizes unmethylated CpG-containing DNA motifs (96)]. Activation of these TLRs, with the exception of TLR3, incorporates MyD88 to the respective receptor complex which subsequently interacts with TRAF6 leading to nuclear translocation of NFkB (97) and type-I IFN induction (98). TLR3 alternatively signals *via* the adaptor TRIF which activates TBK1 and subsequently leads to type-I IFN induction *via* phosphorylation of IRF3 (97). Furthermore, TLR3 signaling can activate cell death cascades by engaging RIP1 and RIP3 (99). Keratinocytes, constitutively express TLR 3 and 9 and their stimulation with corresponding ligands results in induction of TNFa and type-I IFN as well as ICAM1 (100). Interestingly, in oral LP, induction of TLR9 has been described (101). Although keratinocytes do not constitutively express TLR7 or 8, several case reports describe individuals who have developed LP and LS upon use of Imiquimod, an agonist of TLR7/8 (102–104) which is possibly explained by keratinocytes expressing TLR7 under specific conditions (105).

## An *In Vitro* Model to Study Interface Dermatitis

### Background

In 2016, our working group has first established an *in vitro* model that mirrors our understanding of ID as being fueled by endogenous nucleic acids (106) and further characterized it within a study from 2017 (50). Stimulation with eNA results in a pronounced expression of typical ID-associated cytokines within different keratinocyte models. IFNy, mainly produced by lymphocytes, is known to play a pivotal role in the pathogenesis of diseases featuring ID (107–109), and has been shown to induce typical morphological changes in human epidermis equivalents, *in vitro* (110). Herein, we aim to deliver an in-depth analysis of differentially regulated genes in our ID model and furthermore present synergistic effects of endogenous nucleic acids in addition to IFNy on human keratinocytes.

### Results

#### Cytosolic Localization of DNA Motifs in Interface Dermatitis Keratinocytes

**Figure 1:**
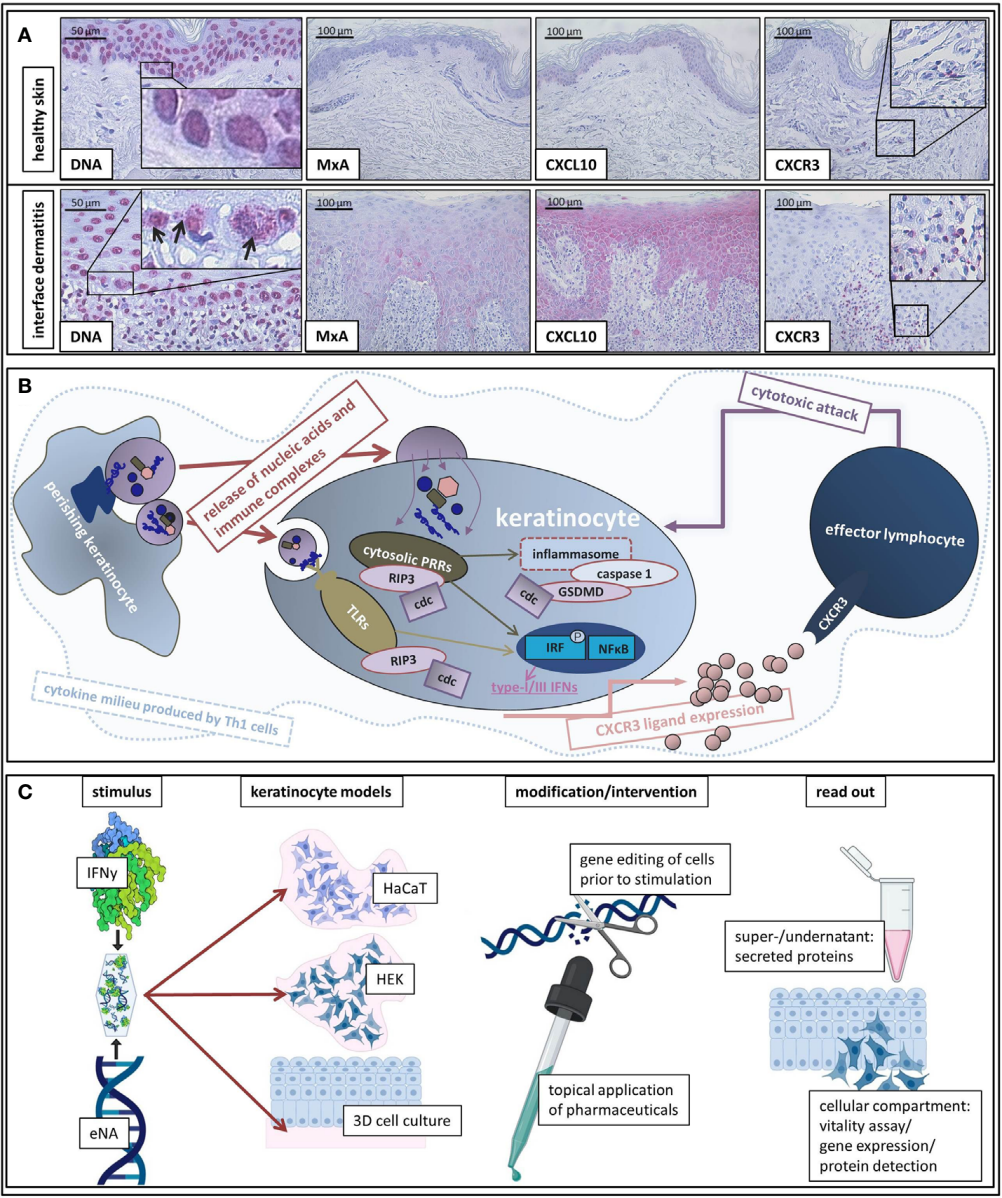
**(A)** Representative histological findings in interface dermatitis and healthy skin. Representative findings of DNA, MxA, CXCL10, and CXCR3 immunostaining in healthy skin and interface dermatitis (lichen planus). Original magnification x200 (x400 concerning DNA). Black arrows highlight extranuclear localization of DNA motifs. Boxes highlight digitally enlarged aspects. **(B)** Schematic presentation of assumed etiopathological mechanisms of interface dermatitis (as reviewed above). ID is characterized by a Th1 cytokine milieu in which endogenous nucleic acids activate PRRs. Downstream signaling unleashes cell death cascades (cdc) and leads to production of type-I and -III IFNs and pro-inflammatory cytokines as well as inflammasome activation. Interferon-inducible chemokines (produced by keratinocytes upon autocrine IFN-stimulation) recruit CXCR3 + effector cells into lesional skin, which induce keratinocyte perishing and thus release of pro-inflammatory cell components. **(C)** Schematic presentation of our *in vitro* model of interface dermatitis. Nucleic acids extracted from keratinocytes and IFNy are administered to different keratinocyte models (HaCaT, HEK, epiCS) as an ID-like stimulus. Genetic modification of the cells of interest can be made prior to stimulation in order to evaluate specific components of ID pathogenesis. Furthermore, the effect of innovative pharmaceuticals on ID-like stimulation can be analyzed. Super-/undernatants and the cellular compartment are available to read out methods.

DNA motifs in extranuclear compartments were significantly more present in ID than in healthy control samples (**Figure 1A**).

#### CXCL10 and MxA are Expressed by Keratinocytes in Interface Dermatitis and the Majority of Infiltrating Immune Cells Express CXCR3 Receptors

**Figure 1A** depicts findings within a LP skin specimen that are representative for all examined samples: MxA (MX Dynamin Like GTPase A) and CXCL10 are expressed by keratinocytes and the majority of infiltrating immune cells carries CXCR3 receptors.

#### Stimulation With Endogenous Nucleic Acids Induces a Molecular Signature in Keratinocytes Resembling Interface Dermatitis

**Figure 2:**
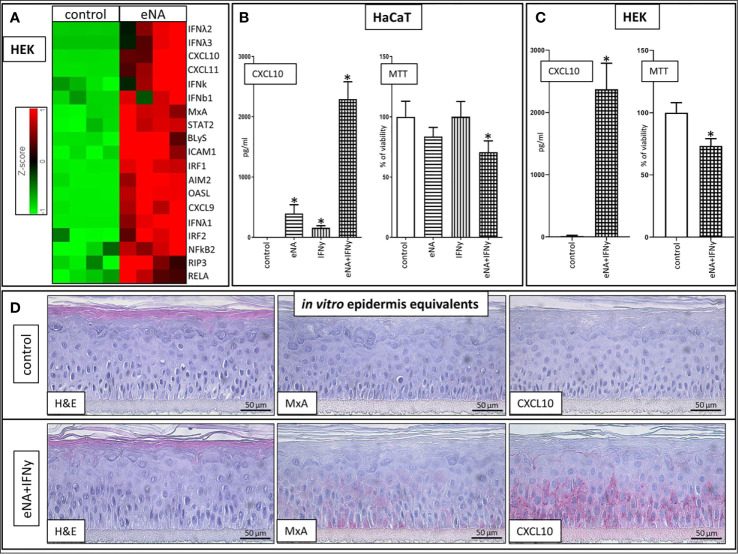
**(A)** Expression of upregulated genes involved in ID pathogenesis in HEK cells stimulated with eNA (12,5 µg/ml) for 24 h compared to HEK cells solely exposed to medium (control), (n = 4, fold change > 2, p < 0.01, Partek^®^ Flow^®^). **(B)** CXCL10 levels within the supernatant of HaCaT cells stimulated with eNA (5 µg/ml) or IFNy (1 x 10^3 U/ml) or the combination of both for 20 h compared to HaCaT cells solely exposed to medium (control), MTT assay executed on the cells corresponding with the respective supernatant, mean of controls defined as 100%. Given are respective means with standard deviations indicated by error bars (n = 4, * indicates significance (p < 0.05), Mann Whitney test). **(C)** CXCL10 levels within the supernatant of HEK cells stimulated with the combination of eNA (5 µg/ml) and IFNy (1 x 10^3 U/ml) for 6.5 h compared to HEK cells solely exposed to medium (control), MTT assay executed on the cells corresponding with the respective supernatant, mean of controls defined as 100%. Given are respective means with standard deviations indicated by error bars (n = 4, * indicates significance (p < 0.05), Mann Whitney test). **(D)** Representative findings in 3D epidermis equivalents upon control (medium) settings and stimulation with eNA (5 µg/ml) and IFNy (1 x 10^3 U/ml) for 22 h. Hematoxylin and eosin stain, MxA, CXCL10. Original magnification ×400. (n = 3).

In normal human epidermal keratinocytes (HEK), stimulation with eNA significantly induces expression of genes encoding key drivers (111–117) of innate inflammatory pathways (IRFs, IFNs, STAT2, RELA, NFkB, CXCL9/10/11, Mx1, OASL), inflammasome activation (AIM2), cell death (RIP3) and factors mediating interaction between keratinocytes and T cells (ICAM1) as well as the adaptive immune system (BLyS) (**Figure 2A**).

#### Stimulation With IFNy and Endogenous Nucleic Acids Induce CXCL10 and MxA Expression

Stimulation with IFNy or eNA respectively leads to significant expression of CXCL10 in HaCaT cells. Combined administration of IFNy and eNA results in an over-additive effect concerning CXCL10 release (**Figure 2B**). Strong CXCL10 expression upon concomitant stimulation could be confirmed in normal human epidermal keratinocytes (HEK, **Figure 2C**) and in reconstructed human epidermis equivalents (epiCS, **Figure 2D**). Furthermore, MxA expression is induced upon combined stimulation in epiCS whose staining resembles the pattern detected in ID (compare **Figure 1A**).

#### Concomitant Stimulation with IFNy and Endogenous Nucleic Acids Has a Direct Cytotoxic Effect on Keratinocytes

Cytotoxic effects in our approach were not significant upon stimulation with eNA or IFNy alone. Upon concomitant stimulation, however, the ability to reduce MTT reagent into its insoluble formazan was significantly impaired in both HaCaT (**Figure 2B**) and HEK (**Figure 2C**) serving as a marker for cell viability.

### Methods

Please find a description of applied methods as a supplement to this main text.

## Discussion

Our working group has previously described extranuclear DNA motifs being significantly more present in keratinocytes of CLE patients than in healthy skin (50). We herein present analogous findings in LP patients. *In vitro*, stimulation with endogenous nucleic acids induces a gene expression pattern in human keratinocytes which resembles key features of ID: We present IFNλ induction upon stimulation with eNA in keratinocytes, which is known to be significantly elevated in skin diseases featuring ID but neither in healthy controls nor other inflammatory skin diseases (22). The type-I IFNs, IFNb and IFNk, are both induced in keratinocytes upon stimulation with eNA. IFNb has been described to be expressed in basal epidermal layers of LP (118) skin. IFNk has been shown to be highly expressed in CLE (19) and LP skin but not in other inflammatory dermatoses (119) and is acknowledged to be a key regulator of IFN response in keratinocytes. Stimulation with eNA did not upregulate expression of IFNa. Although it has been detected in keratinocytes of the whole epidermis in LP skin (118), pDCs are considered to be the main producers of IFNa *in vivo* (17, 36, 120). Via autocrine loops, all type-I IFNs bind to IFNAR (23, 24, 121) and type-III IFNs signal *via* their receptor IFNLR (24). Activation of both receptors causes phosphorylation of JAK1 and TYK2 (25). Receptor bound STATs (STAT1 and STAT2) are subsequently phosphorylated leading to heterodimerization and formation of ISGF3 together with IRF9 (122). This complex translocates to the nucleus and induces expression of genes that exhibit specific ISREs (Interferon-sensitive response elements) to which the complex binds. Amongst such genes are OAS, MxA and multiple transcription factors, including IRFs (24, 38) which are induced upon eNA-stimulation in our experiments. While IFNAR is expressed on nearly all cell types and IFNLR is mainly restricted to epithelial cells, their downstream signaling is quite congruent (24). Type-I IFN dependency (24), however, is described for ISGF3-like complex formation, which consists of IRF9 and STAT2 homodimers and can reinstate inflammatory cascades in the absence of STAT1 (123).

In our approach, type-II IFN was not induced in keratinocytes upon eNA-stimulation, which is in accordance with the view that the pronounced presence of type-II IFN (IFNy) in ID skin derives from other sources than keratinocytes. Specifically, it is predominantly produced by natural killer cells, group 1 innate lymphoid cells, yδ T cells and CD8+ cytotoxic T cells as well as CD4+ Th1 cells [as reviewed in (124)]. Its receptor (IFNyR) signals *via* the JAK1/JAK2 and STAT1/STAT2 pathway (107, 125). Shao et al. describe that, *in vitro*, priming of keratinocytes with type-I IFNs, and to an even greater extent type-II IFNs, increases their susceptibility to MHC I-dependent, T-cell mediated cytotoxicity (107). Knock out of JAK2 or STAT1 inhibited this induction of MHC I in keratinocytes upon IFNy-stimulation whereas only minimal suppression was detected in JAK1 or STAT2 KO cells (107). The potential of human keratinocytes as nonprofessional antigen-presenting cells has recently been further underlined by Orlik et al. who have demonstrated the capacity of IFNy-pretreated keratinocytes to activate co-cultured naïve T-cells (126). ICAM1 is a further mediator supporting interaction between T lymphocytes and keratinocytes that is inducible by IFNy (127) and is overexpressed in diseases featuring ID (128). Our data, in turn, shows that ICAM1 is also induced upon stimulation with eNA. Furthermore, cell death cannot only be induced *via* activation of infiltrating immune cells but also *via* induction of keratinocytic apoptosis (cleaved caspase 3) and necroptosis (RIP3) as both factors have been shown to be overexpressed in keratinocytes of LP and CLE patients (16, 107). These markers can be induced by IFNy (107) and although stimulation with eNAs alone does not result in a significant reduction of cell viability as measured by vitality assay, cell death cascades are activated upon stimulation with eNA that mimic the ones described in ID as we detected induction of RIP3 and the inflammasome component AIM2. Furthermore, significant cytotoxicity is detectable upon concomitant stimulation of keratinocytes with eNA and IFNy. A cross-talk by keratinocytes to the adaptive immune system is mediated by BLyS, a B lymphocyte survival factor (129) which has been described to be overexpressed in CLE and LP (130). We herein show that it is induced upon stimulation with eNA. Another mediator implicated in immune and inflammatory responses is NFkB that has been shown to be among the top regulated genes shared by LP and CLE (16). This crucial transcriptional factor family comprises NFkB1, NFkB2, RELA, RELB, and C-REL (131): Stimulation with eNA induces this important mediator in keratinocytes. As reviewed above, expression of CXCR3 ligands by lesional keratinocytes is decisive for attraction of effector cytotoxic T cells. CXCL10 (26, 27) and CXCL11 (28) have been shown to be inducible by type-I interferons. CXCL9, on the other hand, has repetitively been described as truly dependent on IFNy (26, 132). Our group, however, has demonstrated CXCL9 induction in keratinocytes upon stimulation with the type-I interferon IFNk earlier (22). Furthermore, a recent study has detected CXCL9 expression in keratinocytes as a result of inflammasome activation (133). As outlined above, inflammasome activation is another major pathway upon sensing of nucleic acids that might explain expression of all three CXCR3 ligands by stimulation with eNA in the absence of externally administered IFNy.

Stimulation of keratinocytes with endogenous nucleic acids induces key mediators of ID. According to the mechanisms discussed above, we are convinced that addition of IFNy to the interface dermatitis model promotes an even more realistic imitation of *in vivo* scenarios. Concomitant stimulation of keratinocytes with endogenous nucleic acids and IFNy not only promoted direct cytotoxicity but also caused an overadditive effect on CXCL10 level elevation.

## Outlook

Lichen planus as well as cutaneous lupus erythematosus go along with a high disease burden and are considered therapeutically challenging because current treatments often fail to achieve disease control (134–136). We are convinced that preclinical studies and clinical trials evaluating innovative future therapeutic approaches should not focus on one particular condition but rather on clusters of diseases featuring common immune response patterns. Our working group has recently successfully employed the here described model to elucidate the influence of JAK inhibition on keratinocytes in an interface-dermatitis-like context (121). In the herein described refined version of the model IFNy mimics the presence of a T-helper cell mediated cytokine milieu and together with eNA synergistically intensifies the resulting pro-inflammatory signature. Our model represents pathomechanistic key features of ID and thus enables evaluation of potential future pharmaceuticals. It might aid in predicting therapeutic response to novel treatment strategies in therapeutically challenging diseases featuring ID.

## Data Availability Statement

The datasets generated for this study are available on request to the corresponding author.

## Ethics Statement

The studies involving human participants were reviewed and approved by the Ethics Committee of the Medical Faculty of the University of Bonn, Venusberg-Campus 1, 53127 Bonn. The patients/participants provided their written informed consent to participate in this study.

## Author Contributions

CB designed the study. CB, LD, and TF performed the experiments. CB and JW analyzed the data. DN and TB contributed essential resources during manuscript preparation. All authors contributed to the article and approved the submitted version.

## Funding

CB receives a scholarship from the Medical Faculty of the University of Bonn (“Gerok-scholarship”, grant numbers 2019-1A-02 and 2020-1A-07).

## Conflict of Interest

The authors declare that the research was conducted in the absence of any commercial or financial relationships that could be construed as a potential conflict of interest.

## Abbreviations

AIM2, Absent In Melanoma 2; cGAS, Cyclic GMP-AMP synthase; CLE, Cutaneous lupus erythematosus; DAMP, Damage associated molecular patterns; DM, Dermatomyositis; ds, double stranded; eNA, endogenous nucleic acids; ID, Interface dermatitis; IFI16, Gamma-interferon-inducible protein 16; IFNa, Interferon alpha; IFNb, Interferon beta; IFNy, Interferon gamma; IFNk, Interferon kappa; IFN λ, Interferon lambda; IRF1/2/3, Interferon Regulatory Factor 1/2/3; ISREs, Interferon-sensitive response elements; LGP2, (Laboratory of Genetics and Physiology 2); LP, Lichen planus; LS, Lichen sclerosus; MDA5, Melanoma differentiation-associated protein 5; MxA, MX Dynamin Like GTPase A; PAMP, Pathogen associated molecular pattern; pDC, plasmacytoid dendritic cell; PRR, Pattern recognition receptors; RLR, RIG-I-like receptors; SLE, Systemic lupus erythematosus; ss, single stranded; STING, Stimulator of IFN genes; TBK1, TANK-binding kinase 1; TLR, Toll Like Receptor; ZBP1, Z-DNA Binding Protein 1.
