# Supplementary material for: Immunostimulatory Endogenous Nucleic Acids Perpetuate Interface Dermatitis—Translation of Pathogenic Fundamentals Into an In Vitro Model
Source: Front Immunol. 2021 Jan 11;11:622511. doi: 10.3389/fimmu.2020.622511 (PMC7831152; doi:10.3389/fimmu.2020.622511)
Supplement: Supplementary file 1 [file DataSheet_1.docx]

**Methods**

**Skin samples and immunohistochemistry**

Sections from LP patients (n=7) and CLE patients (n=5) were prepared from formalin-fixed, paraffin-embedded skin biopsies. Immunohistochemistry was performed using the following primary antibodies: anti-DNA (AC-30-10, BIOZOL, Eching, Germany), anti-MxA (M143, Prof. Otto Haller, Freiburg, Germany), anti-CXCL10 (abcam, Cambridge, UK), anti-CXCR3 (BD Biosciences, San Jose, USA).

**Cell culture and stimulation**

Immortalized keratinocytes (HaCaT) derived from CLS Cell Lines Service GmbH (Eppelheim, Germany), normal human epidermal keratinocytes (HEK) from CellSystems (Troisdorf, Germany). Cell lines were cultured according to standard protocols. Cultured keratinocytes were stimulated with respective endogenous nucleic acids isolated from either unstimulated HaCaT or HEK using the “Genomic DNA from tissue” kit from Macherey-Nagel (Dueren, Germany). No RNA digestion was performed. DNA concentration within the resulting eluted products were measured via NanoDrop 1000 Spectrophotometer (Thermo Fisher Scientific) and eNA were applied in a concentration of 5 μg DNA/ml (Figure 2B, 2C and 2D) or 12,5 µg DNA/ml (Figure 2A). Concerning the data depicted in Figure 2A and 2D Lipofectamine 2000 served as transfectant (2,5 μl/ml, Invitrogen, Carlsbad, CA, United States). FuGENE (10µl/ml, Promega Corporation, Fitchburg, USA) functioned as transfection reagent in the experiments described in Figure 2B and 2C. IFNy (PeproTech GmbH, Hamburg, Germany) was administered in a concentration of 1x10^3 U/ml.

**Read out supernatant - ELISA**

Supernatants were harvested and analyzed concerning their CXCL10 concentration via the human CXCL10/IP-10 DuoSet ELISA from R&D systems (Minneapolis, USA) and using Bio-tek Synergy™ HT Multi-Detection Microplate Reader and the Gen5 (V1.11.5) software.

**Read out cellular compartment**

Cells were either processed for MTT assay procedure or RNA-isolation for gene expression analysis.

**Vitality assay**

After removing the supernatant, cells were exposed to 10% MTT reagent-containing medium for 3.5h (Thiazolylblau, Carl Roth GmbH + Co. KG, Karlsruhe, Germany, dissolved in PBS (5mg/ml)). After supernatant removal, formazan precipitates were solubilized by DMSO addition and its absorbance was measured via the aforementioned Microplate Reader.

**RNA isolation and gene expression analysis**

RNA was isolated using the NucleoSpin RNA kit (MACHEREY-NAGEL, Düren, Germany) according to the manufacturer’s instructions. Samples were further processed by the next generation sequencing (NGS) Core Facility of the Medical Faculty of the University of Bonn.

**3D epidermis equivalents**

Human epidermis equivalents (epiCS) were purchased from CellSystems (Troisdorf, Germany). After stimulation with eNA isolated from HEK cells for 22h, that was initiated at day 14 after air-lift and conducted analogously to the described monolayer cell approaches, epiCS were formalin fixed for 1h, paraffin embedded and then processed for immunohistochemistry as described above.

**Statistics**

Statistical analysis was performed using Partek® Flow® and GraphPad PRISM 8 software.
